# Incident duration prediction through integration of uncertainty and risk factor evaluation: A San Francisco incidents case study

**DOI:** 10.1371/journal.pone.0316289

**Published:** 2025-01-02

**Authors:** Amirreza Salehi, Ardavan Babaei, Majid Khedmati

**Affiliations:** 1 Department of Industrial Engineering, Sharif University of Technology, Tehran, Iran; 2 Faculty of Industrial Engineering, K. N. Toosi University of Technology, Tehran, Iran; 3 Department of Industrial Engineering, Istinye University, Istanbul, Turkey; Sichuan University, CHINA

## Abstract

Predicting incident duration and understanding incident types are essential in traffic management for resource optimization and disruption minimization. Precise predictions enable the efficient deployment of response teams and strategic traffic rerouting, leading to reduced congestion and enhanced safety. Furthermore, an in-depth understanding of incident types helps in implementing preventive measures and formulating strategies to alleviate their influence on road networks. In this paper, we present a comprehensive framework for accurately predicting incident duration, with a particular emphasis on the critical role of street conditions and locations as major incident triggers. To demonstrate the effectiveness of our framework, we performed an in-depth case study using a dataset from San Francisco. We introduce a novel feature called "Risk" derived from the Risk Priority Number (RPN) concept, highlighting the significance of the incident location in both incident occurrence and prediction. Additionally, we propose a refined incident categorization through fuzzy clustering methods, delineating a unique policy for identifying boundary clusters that necessitate further modeling and testing under varying scenarios. Each cluster undergoes a Multiple Criteria Decision-Making (MCDM) process to gain deeper insights into their distinctions and provide valuable managerial insights. Finally, we employ both traditional Machine Learning (ML) and Deep Learning (DL) models to perform classification and regression tasks. Specifically, incidents residing in boundary clusters are predicted utilizing the scenarios outlined in this study. Through a rigorous analysis of feature importance using top-performing predictive models, we identify the "Risk" factor as a critical determinant of incident duration. Moreover, variables such as distance, humidity, and hour demonstrate significant influence, further enhancing the predictive power of the proposed model.

## Introduction

Traffic congestion represents a widespread challenge experienced in urban settings, marked by a significant reduction in the speed or even a complete standstill of vehicular traffic within the road infrastructure. Traffic congestion is exacerbated by rapid economic growth, which attracts an increasing number of people to urban areas in search of improved access to education, housing, and job markets [1]. Traffic congestion can be categorized into recurrent, occurring during peak hours due to high demand, and non-recurrent, resulting from unpredictable events like incidents, weather, or protests. Non-recurrent incidents are the leading causes of traffic congestion [2]. An incident in traffic context refers to any unplanned event or disruption, such as a breakdown or road closure, that can cause congestion. An accident in traffic involves collisions or crashes resulting in damage or injury. Car accidents, as the most important contributing factor in non-recurrent traffic congestion, are thoroughly investigated in our paper. This challenge leads to time wastage, economic losses for travelers, and gives rise to numerous social and environmental concerns [3]. According to the report, on average, Americans spent 99 hours per year stuck in traffic, resulting in a total cost of nearly $88 billion in 2019, equating to an average expense of $1,377 annually per person [4]. Incident management involves the coordination of non-recurrent events, such as incidents, breakdowns, and unusual congestion, which can have a significant impact on travel, safety, the environment, and related costs [5]. Incident management programs are established with the primary goal of minimizing the negative impact of incidents on traffic congestion. These programs are designed to swiftly restore the full capacity of a road network following an incident, thus reducing delays for commuters and travelers. Understanding incident characteristics and patterns through systematic data collection and analysis is vital to minimize their impact on traffic congestion [6]. Comprehending the various factors that impact the duration of traffic incidents holds significant importance for enhancing traffic incident management. To effectively mitigate traffic impacts, this understanding allows for the strategic deployment of resources and personnel. Precise prediction of incident duration is essential for improving travel time reliability and providing dependable traffic information to the public [7, 8]. The remaining sections of the paper are structured as follows: Literature Review section offers a comprehensive review of the relevant literature. Requirements and Methodology section details the methodology, including requirements and the proposed framework. Results section presents an in-depth analysis of the results from both Machine Learning (ML) classification and regression, alongside the findings from the Multiple Criteria Decision-Making (MCDM) process. Discussion section discusses the implications and insights related to the proposed framework. Finally, last section concludes the paper with a summary of key findings, future research directions, and an overview of study limitations.

## Literature review

In the domain of traffic incident management, accurately predicting the duration of incidents stands as a crucial factor in efficiently managing traffic congestion. As highlighted in the preceding section, discerning the underlying factors influencing incident duration and developing predictive models are crucial for advancing traffic management strategies. Consequently, previous studies can be classified into two categories: investigating incident duration factors and predicting incident duration [9]. Investigating incident duration factors studies involve in-depth research into the various factors and variables influencing the duration of traffic incidents. These studies encompass the analysis of historical data, information collection from incidents, and the application of statistical or data-driven methods to identify the key factors of incident duration.

In contrast, predicting incident duration studies concentrate on creating predictive models capable of estimating real-time or near-future traffic incident durations. The fundamental difference between these two categories can be found in their primary objectives. The first category seeks to investigate the factors that impact the duration of incidents, laying the groundwork for understanding the reasons behind variations in incident duration. In contrast, the second category is concentrated on practical implementation, utilizing this knowledge to create predictive tools aimed at reducing the effects of incidents on traffic flow and enhancing overall traffic management. In this regard, a detailed overview of research efforts in each of these areas will be provided.

### Investigating incident duration factors

N. Islam et al. [10] investigated the effect of various factors on freeway incident clearance times in Alabama using advanced econometric models, with the latent class model proving to be the superior fit. The findings aim to inform policies for reducing freeway incident durations and guide evidence-based policy decisions to mitigate traffic disruptions. H. J. Haule et al. [11] presented the concept of "incident impact duration" as a novel performance metric for transportation agencies’ incident management programs. It considers factors influencing this duration, including incident detection methods and emergency services, highlighting its enhanced predictive accuracy compared to the conventional "incident clearance duration". X. Su et al. [12] investigated the influence of weather conditions on traffic delays stemming from incidents in New York State during 2020. Notably, strong winds and reduced visibility extend delay durations, while higher temperatures and strong breezes intensify delays. The study highlights that wind speed, temperature, and visibility are key factors impacting delay severity, offering valuable insights for tailored traffic management strategies. Q. Luo and C. Liu [13] conducted a comprehensive study, exploring the factors impacting the duration of road closures following tunnel traffic incidents in Pennsylvania from 1997 to 2020. The aim was to uncover distinct patterns and attributes, laying the groundwork for effective approaches to minimize the duration of road closures.

### Predicting incident duration

A. Grigorev et al. [2] developed a bi-level ML framework with outlier removal and intra-extra joint optimization to predict incident duration and determine the optimal threshold for classifying short-term and long-term traffic incidents. This research aimed to enhance incident duration prediction for improved route choice and traffic management. R. Rahman and S. Hasan [14] presented a novel data-driven method involving a dynamic graph convolutional long short-term memory neural network model to forecast evacuation traffic in hurricane scenarios. The study aimed to improve evacuation traffic management strategies by tackling the complexities of real-time prediction and uncertainties associated with evacuation behavior. L. Li et al. [15] developed a deep fusion model to improve the accuracy of traffic incident duration prediction by incorporating both incident characteristics and spatial-temporal correlations in traffic flow, surpassing prior models and highlighting the significance of feature fusion in the analysis of traffic incident data. B. Wang et al. [16] introduced a high-dimensional distributional prediction (HDP) framework to address the limitations of existing neural network models in short-term traffic prediction. This framework aimed to provide adaptive distributional predictions for quantifying uncertainty and improve point prediction accuracy and robustness in various traffic conditions. Li et al. [17] proposed a competing risks mixture model to analyze traffic incident duration, considering clearance methods and covariates. Regression analysis on Singaporean expressway data showed significant impacts of traffic conditions and incident characteristics on both clearance methods and incident duration. The mixture model outperformed traditional models, predicting incident duration with reasonable accuracy. Tran et al. [18] proposed a Multi-structured Graph Neural Network (MSGNN) for network-wide incident prediction, leveraging spatiotemporal relationships among links within sub-areas. This model outperforms benchmarks, offering superior performance across various prediction horizons and study networks. Its multi-structured graph input architecture enhances accuracy by fusing heterogeneous data sources effectively.

### Research gaps and contribution

Given the pivotal role of predicting incident duration in traffic management systems, the existing literature presents several approaches for this task. Despite these efforts, several challenges and unresolved questions remain in the precise estimation of traffic incident durations. Specifically, this study identifies the following research gaps:

- The previous studies have often overlooked the risk of incidents related to specific street features in Machine Learning (ML) models. This omission leads to a gap in accurately predicting incident duration by not considering the risk factors associated with different streets.
- Many prior studies have relied on the Federal Highway Administration’s (FHWA) definition of traffic incidents, classifying them primarily based on duration. This rigid categorization does not fully capture the complexities and variations of incidents across different datasets and locations, potentially leading to less effective solutions.
- A significant gap in the literature is the lack of attention to how different incidents impact the traffic flow. This gap limits the effectiveness of models in predicting the true consequences of incidents on traffic management.
- The previous studies on traffic incident duration prediction have often focused on modeling incidents within clearly defined clusters. However, a significant gap exists in the accurate prediction of incidents that fall into boundary clusters, where the characteristics overlap between multiple main clusters. The traditional methods have not adequately addressed the challenges posed by these boundary clusters, leading to less accurate predictions and suboptimal resource allocation during incident management.

To bridge these gaps, our study introduces the following key contributions:

- The approach proposed in this paper addresses the gap of neglected street features by integrating the RPN concept, quantifying the incident risk for each street. This approach enhances the understanding of incident patterns and street-related risk factors, leading to more accurate predictions.
- To address the limitations of rigid incident classification, the proposed approach incorporates clustering techniques that consider more relevant incident features. Unlike previous methods, the proposed approach allows for flexible clustering into a variable number of groups, adapting to diverse dataset characteristics. Applying the fuzzy clustering further enhances the categorization by identifying well-clustered incidents, boundary cases, or noise.
- The Multiple-Criteria Decision-Making (MCDM) techniques, including Fuzzy MCDM, is employed to rank the incident clusters and assess their potential harm to traffic flow. This approach comprehensively addresses the probabilistic nature of incident features, offering a more nuanced understanding of their impact.
- A novel approach that systematically applies scenario-based predictive modeling to boundary clusters is introduced. By analyzing and comparing different models trained on adjacent clusters, we enhance the accuracy of incident duration predictions in these complex boundary areas. This scenario-based approach not only improves the precision of predictions but also offers a flexible framework for adapting to the unique characteristics of incidents that do not fit neatly into predefined categories.

While the field of traffic incident prediction is indeed densely populated with various models and strategies, our study uniquely positions itself by integrating the risk assessment based on specific street features and addressing the complexities of boundary clusters, which are often overlooked in existing research. Unlike prior studies that primarily focus on broad classifications and traditional modeling approaches, our methodology introduces a more nuanced and adaptable framework that captures the probabilistic nature of incident features and enhances the predictive accuracy in complex scenarios. This contribution not only fills the critical gaps in the literature but also offers practical advancements that can significantly improve the traffic incident management systems.

This research presents a comprehensive framework that significantly enhances incident duration prediction, thereby assisting decision-makers in optimizing resource allocation, mitigating economic impacts, and fostering proactive safety planning. Simultaneously, it empowers end-users with real-time information, alleviating frustration and stress, simplifying route selection, and promoting environmentally responsible travel choices.

## Requirements and methodology

In this section, we outline the key components and methodologies integral to our study. We begin by introducing the Davies-Bouldin Index (DBI), a crucial metric for evaluating the quality of clustering, followed by an explanation of the Fuzzy VIKOR approach, which enhances decision-making under uncertainty. Next, we delve into Fuzzy C-Means (FCM), a widely used clustering algorithm that allows for overlapping memberships. Building on these methodologies, we present the proposed framework for traffic incident analysis, supported by a detailed description of the dataset utilized. Finally, we discuss the concept of risk factors, which play a pivotal role in assessing and predicting the impact of traffic incidents.

### Davies-Bouldin Index (DBI)

The DBI [19], a clustering validation metric, assesses clustering quality by computing the average similarity between each cluster and its most similar cluster. Simultaneously, it incorporates cluster dispersion. Its superiority over alternative metrics lies in its easily interpretable nature, where lower values correspond to well-defined clusters. Additionally, it accounts for both cluster compactness and inter-cluster separation, devoid of any assumptions regarding cluster shapes or densities. Consequently, the Davies-Bouldin Index stands as a versatile and robust tool for evaluating clustering performance across diverse datasets. DBI is formally defined as follows [20]:

$$DBI=\frac{1}{k}\sum_{i=1}^{k}{max}_{j\neq i}\{D_{i,j}\}$$ (1)

where *D*_*i*,*j*_ represents the ratio of distances within and between groups for the *i*th and *j*th groups, expressed mathematically as:

$$D_{i,j}=\frac{\left({\bar{d}}_{i}+{\bar{d}}_{j}\right)}{d_{i,j}}$$ (2)

where ${\bar{d}}_{i}$ represents the average Euclidean distance from each data point within the *i*th group to the centroid of that same group, and *d*_*i*,*j*_ denotes the Euclidean distance between the centroids of the *i*th and *j*th groups.

### Fuzzy VIKOR

Fuzzy VIKOR (MCDM using VlseKriterijumska Optimizacija I Kompromisno Resenje) is a decision-making method adapted for situations with uncertain data. It stands out among other approaches by integrating the VIKOR methodology with fuzzy logic, creating an effective tool for tackling intricate decision-making scenarios replete with conflicting criteria. By integrating fuzzy logic into the VIKOR framework, fuzzy VIKOR offers a more adaptable and resilient approach to decision-making, making it the preferred choice when conventional methods fall short in capturing the intricate interplay of complexity and uncertainty inherent in the problem. The detailed steps for implementing fuzzy VIKOR can be found in Appendix B in S1 File [21].

### Fuzzy C-Means (FCM)

Fuzzy clustering is an efficient, unsupervised technique for data analysis and model creation. It allows objects to have partial memberships between 0 and 1, instead of forcing them into distinct classes. The most widely used method for this is the FCM algorithm [22]. FCM assigns each data point a membership grade between 0 and 1 for each cluster center based on their proximity. The closer a data point is to a cluster center, the higher its membership in that cluster. The sum of memberships for each data point adds up to one. After each iteration, memberships and cluster centers are updated following a specific formula [23]. The detailed steps of the FCM algorithm are provided in Appendix A in S1 File.

### Proposed framework

**Fig 1:**
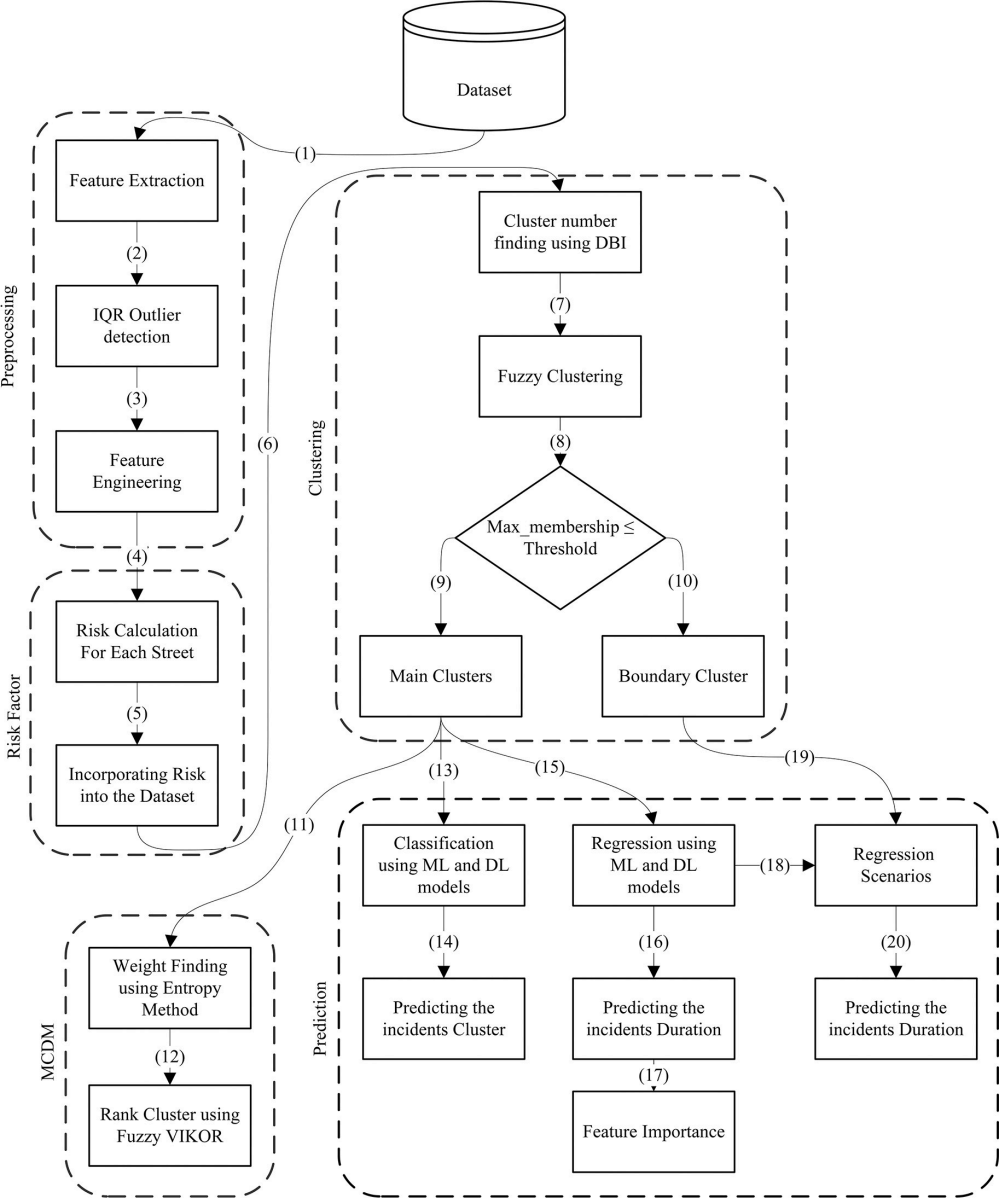
Flowchart of the proposed framework.

As mentioned earlier, traffic congestion is a pervasive problem in urban areas, with a diverse and significant impact on road infrastructure. Incident management programs are designed to restore the full capacity of road networks following incidents by understanding incident characteristics and patterns. To achieve this, it is crucial to understand the factors that influence the duration of traffic incidents and to make accurate predictions of incident durations. In this paper, we present a comprehensive framework designed to efficiently categorize incidents and predict their duration by considering street risk factors. Additionally, we identify and prioritize key features that affect incident durations. Fig 1 illustrates the flowchart of the proposed framework.

According to the flow chart, in the initial step, the San Francisco dataset is derived from the larger “Countrywide Traffic Accident” dataset. In step 1, feature extraction is conducted, and features with over 50% missing values are systematically removed from the dataset. This threshold was chosen based on preliminary analysis, which showed that retaining features with higher missing values negatively impacted model performance. Subsequently, in step two, an IQR outlier detection technique is employed to identify and remove extreme values in the "duration" feature. The IQR method was selected due to its effectiveness in identifying outliers in skewed distributions, which is common in traffic incident data. This removal process improves the prediction accuracy by reducing the impact of both high and low values. In step 3, key features, such as the incident month and time of day, are extracted from the Start_Time and End_Time variables. These variables are selected for their direct impact on predicting the incident duration. For instance, incidents occurring during peak traffic hours tend to have longer durations due to increased congestion. In steps 4 and 5, a comprehensive risk factor is calculated for each street, considering severity, occurrence rate, and detection rate. The risk factor for each street is determined using the RPN concept, which considers the impact of incident location on duration predictions. In step 6, the dataset is subjected to the DBI method to determine the appropriate cluster number. The DBI method is chosen due to its ability to measure the average similarity between each cluster and its most similar cluster, considering the distance between cluster centers. In step 7, fuzzy clustering is used to categorize the incidents based on the acquired data. Fuzzy clustering allows for the assignment of incidents to multiple clusters with varying degrees of membership, reflecting the uncertainty inherent in real-world data. Boundary clusters, which have low membership confidence, represent incidents that do not fit neatly into a single category, making them critical for nuanced analysis. Step 8 introduces a new policy for identifying boundary clusters. The primary criterion for designating an incident as a boundary cluster is if its highest membership percentage falls below a specified threshold of 0.5. Additionally, a secondary condition is applied, where incidents with membership percentages showing minimal differences, typically within a 10% range, are also considered boundary clusters. For instance, if an incident has membership percentages of 45%, 40%, and 15% across three clusters, it is considered a boundary cluster between the first two. If the highest membership percentage for each cluster exceeds the threshold, the incident is not classified as a boundary cluster. Instead, it is assigned to one of the main clusters according to this policy. The main clusters are then ranked using the MCDM process, based on their impact on traffic conditions. While clustering is essential, understanding the differences between each cluster is crucial for effective analysis. To bridge this gap, MCDM approaches use cluster centers as alternative solutions. Clusters with higher scores are generally associated with greater severity and risk compared to other clusters. In step 11, the weight of each cluster center is calculated using the entropy method. The entropy method is used to calculate the weight of each cluster center, reflecting the uncertainty or importance of features within each cluster. Subsequently, in step 12, the fuzzy VIKOR method is employed to rank the cluster centers. In step 13, the incidents within the main clusters are used to build a classification model that employs both Deep Learning (DL) and ML techniques. This classification model is then used to predict the cluster assignment of each incident, determining whether it belongs to the main cluster or a boundary cluster. Step 14 focuses on developing regression models for the main clusters, utilizing both ML and DL techniques. Given the large dataset, we used 55% of the data for training, 20% for validation, and 25% for testing. This split allows us to robustly compare the performance of different algorithms and identify the most effective model for predicting the incident durations. The most influential predictor feature is identified by evaluating the top-performing model. In steps 16 and 17, the models developed for the main clusters are used to predict the boundary clusters and identify the most effective model for this task.

### Dataset description

To illustrate the efficiency of our proposed framework for predicting incident durations and identifying their key features, we utilized the "Countrywide Traffic Accident" dataset (https://doi.org/10.5281/zenodo.13804904) [24]. This extensive dataset covers traffic incidents occurring across all 49 states in the United States and has been consistently updated from February 2016 to March 2023. Its comprehensiveness is attributed to a network of data providers, encompassing multiple APIs that consistently deliver real-time traffic event data. These APIs collect data from various sources, including federal and state transportation departments, law enforcement agencies, traffic cameras, and sensors embedded in road networks. Presently, the dataset boasts an impressive 7.7 million accident records, making it an invaluable resource for examining various types of road incidents. In our quest for a more focused analysis of incident durations, we extracted data specific to San Francisco from this dataset. Due to the presence of a significant number of missing values in certain features, we meticulously selected 31 relevant features and subsequently filtered out rows with incomplete data. This process yielded a dataset comprising 16,986 accidents, which is well-prepared for thorough analysis and modeling. Table 1 provides a description of the features.

**Table 1:** Dataset description for “Countrywide Traffic Accident Dataset”.

Attribute	Description
Source	The origin of the accident data
Severity	A scale from 1 to 4 indicating traffic impact, where 1 means minor (short delay) and 4 means major (long delay)
Start_Time	The local time when the accident began
End_Time	The local time when the accident’s impact on traffic flow ended
Distance(mi)	The length of road affected by the accident
Street	The name of the street where the accident occurred
Airport_Code	The code for the nearest airport-based weather station to the accident location
Temperature(F)	The temperature in Fahrenheit at the accident location
Humidity(%)	The humidity level at the accident location as a percentage
Pressure(in)	The air pressure at the accident location in inches
Visibility(mi)	The visibility in miles at the accident location
Wind_Direction	The direction of the wind at the accident location
Wind_Speed(mph)	The wind speed in miles per hour at the accident location
Weather_Condition	The weather condition at the time of the accident
Amenity	An annotation indicating the presence of an amenity in the vicinity
Bump	An annotation indicating the presence of a speed bump or hump nearby
Crossing	An annotation indicating the presence of a crossing nearby
Give_Way	An annotation indicating the presence of a give-way sign nearby
Junction	An annotation indicating the presence of a junction nearby
No_Exit	An annotation indicating the presence of a "no exit" sign nearby
Railway	An annotation indicating the presence of a railway nearby
Roundabout	An annotation indicating the presence of a roundabout nearby
Station	An annotation indicating the presence of a station nearby
Stop	An annotation indicating the presence of a stop sign nearby
Traffic_Calming	An annotation indicating the presence of traffic calming measures nearby
Traffic_Signal	An annotation indicating the presence of a traffic signal nearby
Turning_Loop	An annotation indicating the presence of a turning loop nearby
Sunrise_Sunset	The period of the day (day or night) based on sunrise and sunset times
Civil_Twilight	The period of the day (day or night) based on civil twilight
Nautical_Twilight	The period of the day (day or night) based on nautical twilight
Astronomical_Twilight	The period of the day (day or night) based on astronomical twiligh

**Fig 2:**
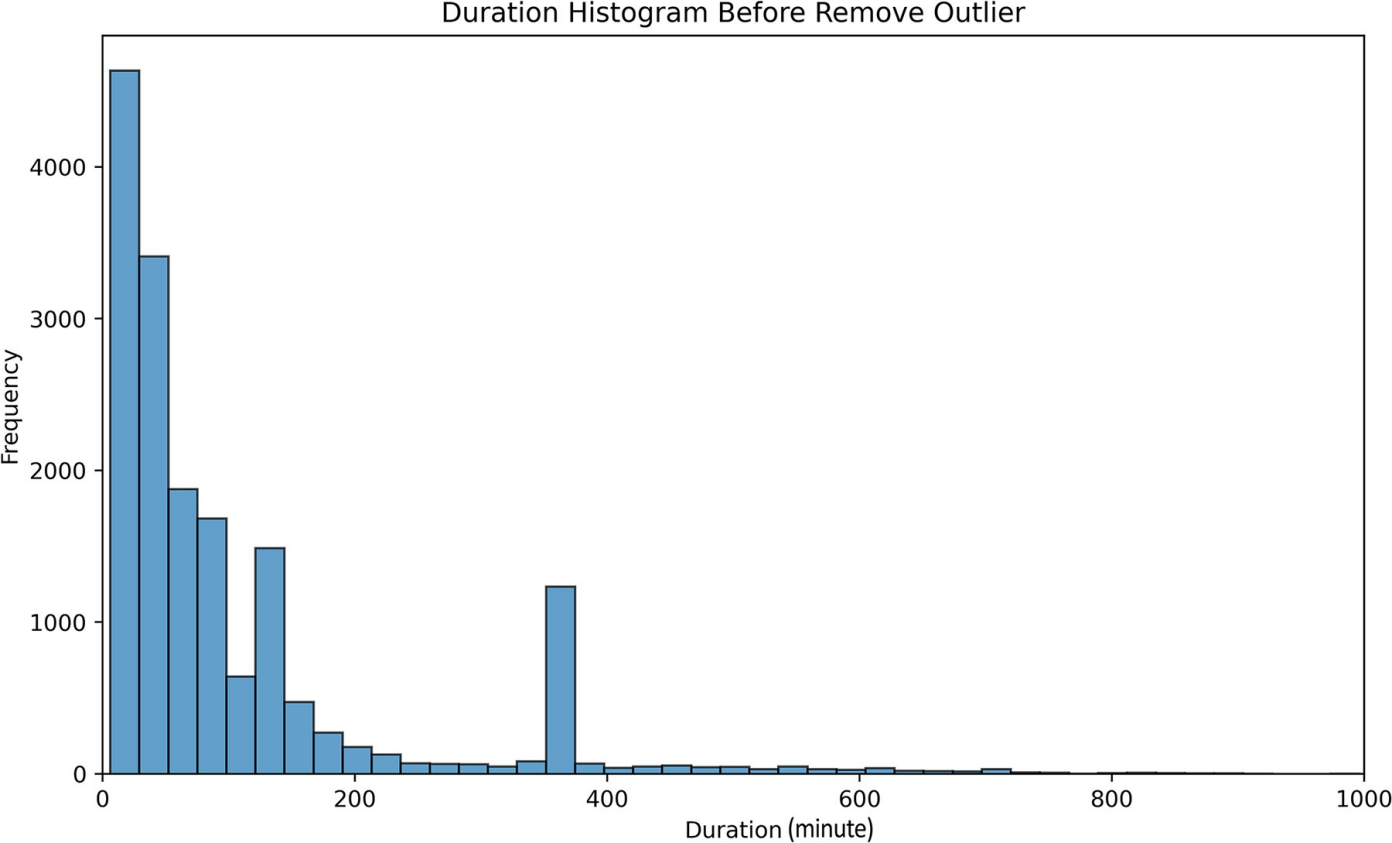
Incident duration distribution before removing outliers.

As previously defined, we determine the incident duration by calculating the time difference between the ’Start_Time’ and ’End_Time’ features. This duration distribution is illustrated in Fig 2. The mean incident duration is approximately 67 minutes, with a standard deviation of 50 minutes, though the distribution shows a right-skewed tail.

**Fig 3:**
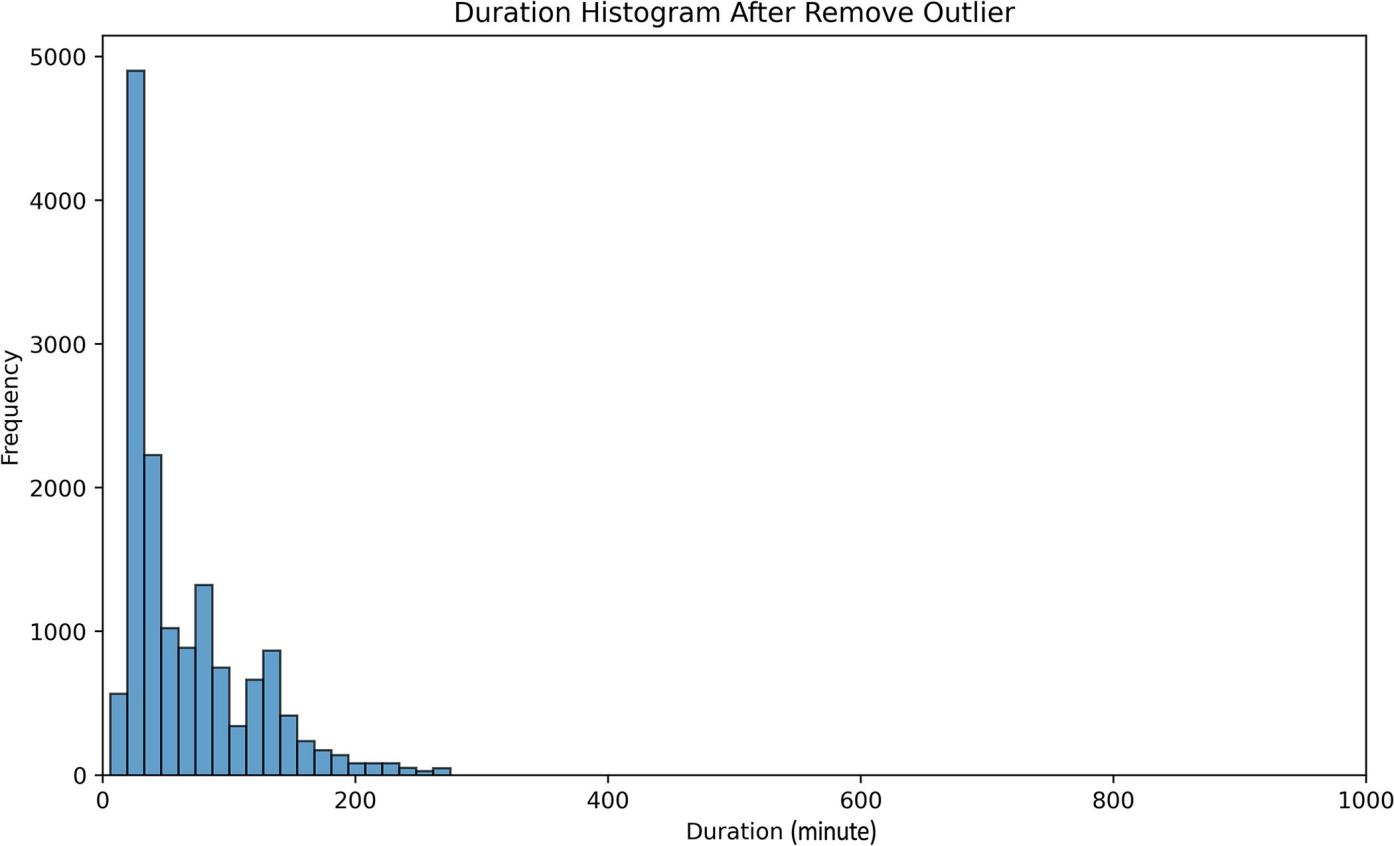
Incident duration distribution after removing outliers.

The extended tail in the duration histogram indicates the presence of outliers within the dataset. To address this issue, we employ the IQR (Interquartile Range) outlier detection method to eliminate these outliers. Fig 3 visualizes the duration distribution after the removal of outliers. Following this cleansing process, the distribution becomes more tightly concentrated around the mean value, resulting in an improved predictive process.

Considering the influence of both the month of the year and the hour of the incident, we derive two essential features from the Start_Time attribute: ’monthidx’ (month index) and ’SHour’ (start hour). Recognizing the varying traffic congestion levels throughout the day, especially during peak hours, understanding the start hour of the incident becomes pivotal. Additionally, analyzing the month of the year offers valuable insights into seasonal patterns, weather-related elements, and Holidays and Special Events factors that significantly impact incident occurrences.

### Risk factor

Risk assessment is a crucial feature in effective traffic management, playing a pivotal role in providing the safety of road users and optimizing the efficiency of transportation systems [25]. Through systematic evaluation of potential risks associated with different aspects of traffic, we can identify, quantify, and mitigate hazards before they escalate into incidents or disrupt the smooth flow of traffic. In this paper, we scrutinize the risks of incidents on each street, aiming to provide comprehensive insights into potential dangers and formulate strategies for enhancing overall safety and operational integrity. An innovative method for evaluating risk in traffic management involves the application of the RPN concept, which has previously been employed in areas like engineering [26] and manufacturing [27] but is now being introduced in the field of traffic management. The RPN approach enables the integration of various factors, including the severity of potential incidents in each street, the frequency of incidents in each street, and the likelihood of detecting incidents in each street, into a unified and comprehensive metric. This approach provides a comprehensive perspective on the risk landscape within our road network. The severity, occurrence rate, and detection rate are calculated for each street using Eqs (3), (4), and (5), respectively.

$$S_{i}=\frac{1}{n}\sum_{j=1}^{n}{Severity}_{i,j},\;\forall i$$ (3)

where *S* is the mean severity of potential incidents for each street, n is the total number of incidents for each street and *Severity*_*i*,*j*_​ represents the severity of the *j*th incident for on street *i*.

$$O_{i}=\frac{1}{T}\sum_{j=1}^{T}a_{ij},\;\forall i$$ (4)

where *O*_*i*_ represents the occurrence rate for street *i*,*a*_*ij*_ denotes the number of incidents on street *i* on day *j* and *T* represents the total number of days.

$$D_{i}=\frac{1}{\frac{1}{n}\sum_{j=1}^{n}{Duration}_{i,j}},\;\forall i$$ (5)

where *D*_*i*_ denotes the detection rate of the *i*th street, *Duration*_*i*,*j*_ represents the duration of the *j*th incident on the *i*th street, and *n* is the total number of incidents on each street. Ultimately, The RPN is calculated using in Eq (6).

$$R_{i}=S_{i}\times O_{i}\times D_{i},\;\forall i$$ (6)

where *R*_*i*_ represents RPN feature for each street.

## Results

In this section, we present the results of our analysis, organized into several key subsections. We begin with a detailed examination of the clustering analysis, highlighting the application of the DBI and FCM methods to categorize the traffic incidents. Then, we explore the evaluation of incident clusters using the Fuzzy VIKOR method, focusing on evaluating and prioritizing incident management strategies. We then transition into the application of various ML models for both classification and regression tasks, comparing their performance in incident classification and duration prediction. This structured approach allows for a comprehensive understanding of the methodologies’ effectiveness and the implications of the results for traffic incident management.

### Clustering analysis

As previously discussed, categorizing incidents based solely on duration is insufficient for effectively distinguishing between them. To address this challenge, the clustering approach is employed to categorize incidents based on all available incident features. The optimal number of clusters is determined using the DBI, which measures the average similarity between each cluster and its nearest cluster, considering the distances between their centers. Based on the results derived from 10 iterations of this cluster determination methodology, it is evident that a 3-cluster configuration is most appropriate. Subsequently, the FCM fuzzy clustering method is employed to assign each incident to a specific cluster and provide cluster membership probabilities for each incident, indicating the likelihood of its association with each cluster. The cluster membership analysis reveals whether an incident belongs to a specific cluster. This classification is valuable for excluding uncertain incidents and identifying suitable models for their prediction. To better illustrate the policy for the main cluster and boundary clusters, Tables 2 and 3 are presented.

**Table 2:** Main clusters illustration.

Severity	Temperature(F)	Humidity(%)	. . .	Cluster_1	Cluster_2	Cluster_3	Cluster
3	71.1	55.0	. . .	0.065470	0.360585	0.573945	3
2	61.0	67.0	. . .	0.033916	0.200471	0.765614	3
3	55.9	80.0	…	0.087320	0.662939	0.249741	2

**Table 3:** Boundary clusters illustration.

Severity	Temperature(F)	Humidity(%)	. . .	Cluster_1	Cluster_2	Cluster_3	Cluster
2	77.0	40.0	. . .	0.125675	0.447762	0.426563	2–3
3	71.1	47.0	. . .	0.101702	0.428778	0.469520	2–3
3	70.0	49.0	…	0.479794	0.395583	0.124623	1–2

In Table 2, each incident has a cluster membership score over 0.5, allowing confident assignment to that cluster. The "…" notation in this table and subsequent tables represents additional variables excluded for conciseness, primarily due to the extensive columns generated by one-hot encoding of categorical variables. In contrast, Table 3 displays incidents where all cluster membership scores fall below 0.5, resulting in two neighboring clusters with membership scores that are very close. For example, in the first incident, the membership scores for Cluster 2 and Cluster 3 differ by about 2%, placing the incident on the boundary between them.

### Fuzzy MCDM cluster evaluation

Employing MCDM on the cluster centers of incidents, considering factors such as humidity, risk, pressure, visibility, wind speed, and various weather conditions, provides the advantage of a structured and systematic approach to evaluating and prioritizing incident management strategies. This method enables a data-driven decision-making process that considers the negative impacts on traffic flow, allowing for more effective and efficient allocation of resources and mitigation efforts within each incident cluster. In incident management, where factors like humidity, risk, pressure, visibility, wind speed, and weather conditions are critical, using Fuzzy MCDM, specifically Fuzzy VIKOR, is crucial. This is because incidents and their related environmental parameters inherently involve uncertainties, vagueness, and imprecision. Fuzzy MCDM excels at managing these complexities, allowing for a more accurate representation of real-world data. Fuzzy VIKOR, within this framework, offers a comprehensive assessment of incident clusters and equips decision-makers to make well-informed, robust traffic management choices. Ranking the cluster centers requires recognizing the criteria that influence traffic flows. To achieve this, we classify these influencing factors into two distinct groups including control criteria and environmental criteria, as outlined in Table 4.

**Table 4:** Types of criteria in traffic management.

Control criteria	Environmental criteria
Amenity	Weather Condition
Bump	Wind_Speed
Crossing	Visibility
Give_Way	Pressure
Risk	Humidity
Junction	
No_Exit	
Railway	
Roundabout	
Station	
Stop	
Traffic_Calming	
Traffic_Signal	
Turning_Loop	

Control criteria are criteria in traffic management that can be controlled or influenced by authorities and regulations. They incorporate aspects such as traffic signals, crossings, and road infrastructure. Environmental criteria encompass uncontrollable factors like weather conditions, visibility, humidity, pressure, and other atmospheric criteria that significantly impact traffic flow. Fuzzy VIKOR scores prioritize incident clusters based on their impact on traffic flow. For instance, a score of 1 for Cluster 3 indicates it is the least problematic, while a score of 0 for Cluster 1 suggests it needs immediate attention and resources. These scores aid decision-makers in effectively allocating resources and guiding intervention strategies, facilitating informed decisions considering various criteria concurrently. Additionally, the scores allow for comparative analysis, enabling benchmarking against previous incidents or across different regions, aiding in continuous improvement by tracking the effectiveness of incident management strategies over time. For instance, an increase in the score of Cluster 1 suggests positive outcomes from the implemented strategies, confirming their effectiveness in improving traffic flow. Table 5 presents the Fuzzy decision matrix, capturing the initial data with fuzzy values, while Table 6 illustrates the decision matrix post-defuzzification, where the fuzzy values have been converted into crisp values, providing a more precise and more interpretable representation of the decision-making process.

**Table 5:** Fuzzy decision matrix.

Humidity(%)	Risk	Pressure(in)	Visibility(mi)	Wind_Speed(mph)	…
(32.0, 73.9069, 100.0)	(0.0, .0092, 0.0188)	(29.16, 30.0094, 30.48)	(0.1, 9.2574, 10.0)	(0.0, 10.6224, 41.0)	…
(15.0, 65.7128, 100.0)	(0.0, 0.0081, 0.0188)	(29.17, 30.0192, 30.58)	(0.25, 9.5728, 10.0)	(0.0, 11.1516, 41.0)	…
(10.0, 66.8924, 100.0)	(0.0, 0.0062, 0.0188)	(29.1, 30.0387, 30.54)	(0.25, 9.4393, 10.0)	(0.0, 10.4238, 48.0)	…

**Table 6:** Decision matrix post-defuzzification.

Humidity(%)	Risk	Pressure(in)	Visibility(mi)	Wind_Speed(mph)	…
68.635634	0.009334	29.883134	6.452468	17.207468	…
60.237601	0.008968	29.923068	6.607601	17.383868	…
58.964134	0.008334	29.892901	6.563101	19.474601	…

According to the inclusion of negative criteria in the ranking of alternatives, Eq (7) is applied as a transformation. This transformation effectively reverses the orientation of the criteria from negative to positive, thereby rendering higher values within the transformed matrix as indicators of superior performance or greater desirability. This change in orientation simplifies the application of decision-making methods or rankings that conventionally operate under the assumption of positive criteria. Consequently, this improves how easily the matrix can be interpreted and used in decision-making.

$$D_{ij}^{\prime }=max_{j}D_{ij}-D_{ij}+1$$ (7)

where $D_{ij}^{\prime }$ is the component in the transformed matrix at the *i*th row and *j*th column, *D*_*ij*_​ is the corresponding component in the original decision matrix and *max*_*j*_*D*_*ij*_ is the maximum value in the *j*th column of original decision matrix.

According to the results of Fuzzy VIKOR, a score of 1.0 in cluster 1 indicates that the incidents mostly occur under conditions that meet both control and environmental criteria. This implies a relatively lower risk associated with these incidents, suggesting that this cluster may require less immediate intervention. However, continuous monitoring and maintenance of control measures are essential to sustain this favorable condition. In cluster 2, with a score of 0.0, there is no discernible alignment with the control and environmental criteria, indicating a need for immediate attention and intervention. These incidents likely arise from conditions not compliant with safety measures, necessitating rigorous control and enforcement actions to enhance the traffic safety in this cluster. Cluster 3, with a score of 0.2739, falls in between clusters 1 and 2, signifying a normal level of alignment with the criteria. This cluster requires attention and improvement but may not be as critical as cluster 2. Efforts should focus on enhancing safety measures and environmental conditions to reduce the risk associated with incidents in this cluster.

### ML models

In this sub-section, we initially classify incidents into two categories: main clusters or boundary clusters. Subsequently, in the regression subsection, we develop regression models designed to predict the incident duration.

#### Classification of incidents

In the classification phase, we employed a range of ML methods, including Artificial Neural Networks (ANN), Random Forest (RF), XGBoost (XGB), K-Nearest Neighbors (KNN), Logistic Regression (LR), LightGBM (LGBM), and Gradient Boosting Decision Trees (GBDT). The dataset is divided into two sets where, 75% is allocated to the training set and 25% to the testing set. The evaluation criteria encompassed Average Recall, Average Precision, and F1-score, as defined by Eqs 8 to 10.

$$Averaged-precision=\frac{1}{C}\times \sum_{i=1}^{C}{Precision}_{i}$$ (8)

where *Precision*_*i*_ represents the precision of *i*th class and C stands for the number of classes.

$$Averaged-recall=\frac{1}{C}\times \sum_{i=1}^{C}{Recall}_{i}$$ (9)

where *Recall*_*i*_ represents the recall of *i*th class and C stands for the number of classes.

$$F1=\frac{1}{C}\times \sum_{i=1}^{C}\frac{(1+\beta^{2}){\times Precision}_{i}\times {Recall}_{i}}{\beta^{2}{\times Precision}_{i}+{Recall}_{i}}$$ (10)

where *Recall*_*i*_ represents the recall of *i*th class, *Precision*_*i*_ signifies the precision of *i*th class, C stands for the number of classes and *β* is employed to balance the significance of precision and recall (supposed to be 1).

Table 7 outlines the ANN configuration, which strategically utilizes a deep architecture with progressively smaller layers to capture complex patterns while preventing overfitting through dropout regularization. The ReLU activation enhances learning efficiency, and the Adam optimizer ensures adaptive learning rates; making the model robust for classification tasks.

**Table 7:** ANN configuration for classification.

Parameter	Value
Layers	(256, 128, 64, 32, 16, 8)
Activation Function	ReLU for hidden layers, Softmax for output
Dropout	0.4 in the first layer, 0.3 in the second
Optimizer	Adam (Learning Rate = 0.001)
Loss Function	Categorical Cross-Entropy
Epochs	150
Batch Size	32

**Fig 4:**
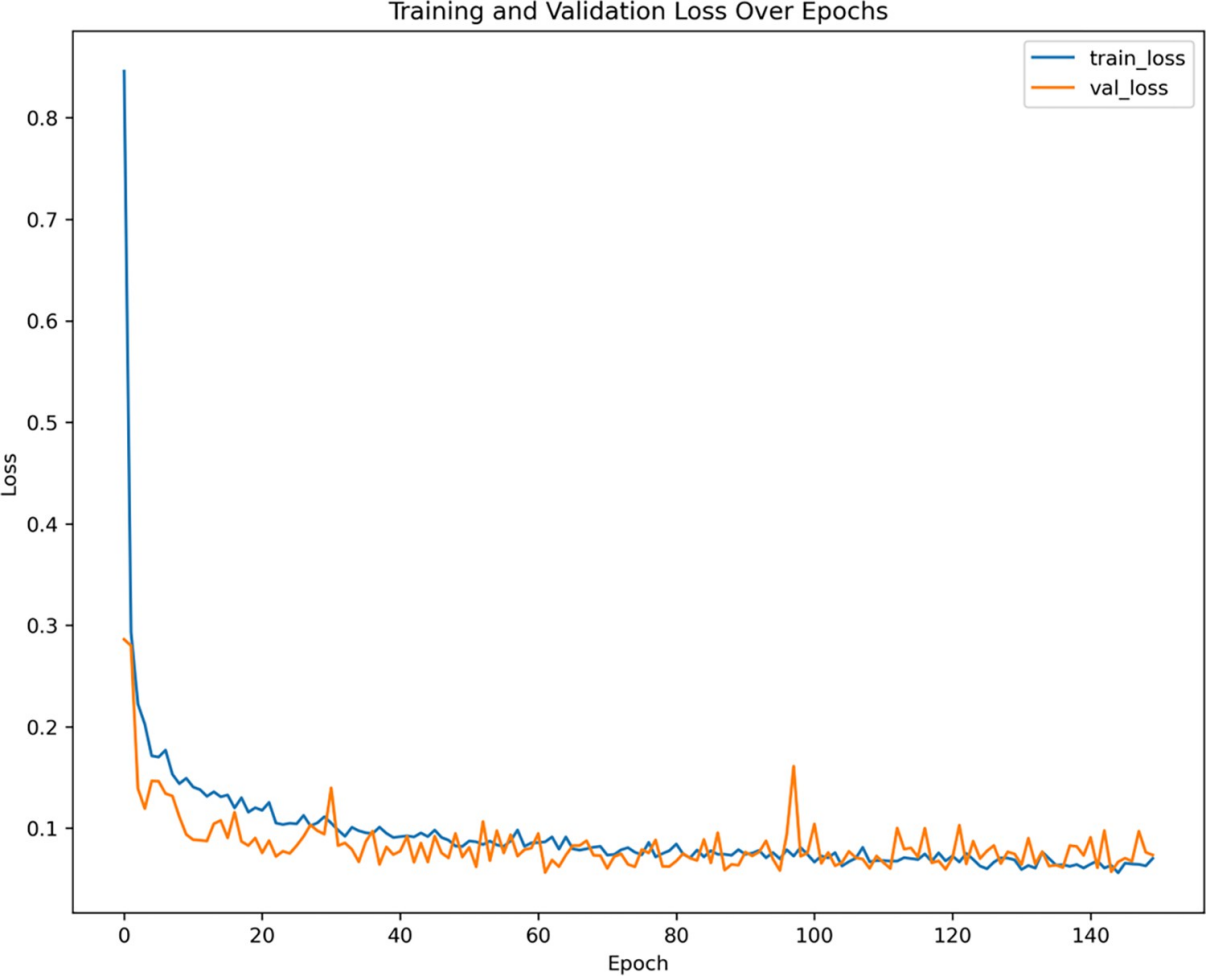
Loss- Epoch plot.

Fig 4 provides a visual representation of the loss per epoch for the ANN model. This plot demonstrates the proficiency of the ANN model in effectively capturing classification patterns and its robust performance in mitigating overfitting.

The performance evaluation, detailed in Table 8, shows that the ANN and LightGBM models led the accuracy metrics, with ANN achieving 97.37% and LightGBM closely following at 97.21%. Regarding Average Recall, ANN also excelled, boasting a score of 0.8726, while XGBoost was the closest competitor with 0.8898. Moreover, ANN demonstrated superior Average Precision at 0.9361, further solidifying its classification performance, with LightGBM closely following at 0.8891. Regarding the F1 Score, ANN outperformed all other models with a score of 0.8995, making it the most effective choice for this classification task. Consequently, ANN emerged as the best-performing model, showcasing its remarkable capacity for incident classification.

**Table 8:** Comparative analysis of ML models for incident classification.

Model	Accuracy	Average Recall	Average Precision	F1 Score
ANN	**0.973**	0.872	**0.936**	**0.899**
RF	0.922	0.644	0.875	0.687
XGB	0.970	0.889	0.885	0.887
KNN	0.946	0.809	0.845	0.824
LR	0.961	0.749	0.752	0.750
LGBM	0.972	**0.890**	0.889	0.888
GBDT	0.959	0.835	0.849	0.841
**Best Model**	**ANN**	**LGBM**	**ANN**	**ANN**

#### Regression for incident duration prediction

For the regression analysis, we utilized a variety of ML algorithms, including RF, XGB, KNN, LR, LGBM, GBDT, and ANN. The ANN was designed with a progressively shrinking layer size to balance complexity and prevent overfitting, as outlined in Table 9. This configuration, featuring ReLU activation and the Adam optimizer, was chosen to maximize learning efficiency and minimize prediction errors using Mean Squared Error as the loss function.

**Table 9:** ANN configuration for regression.

Parameter	Value
Layers	(64, 32, 16, 8, 4)
Activation Function	ReLU
Optimizer	Adam (learning rate = 0.001)
Loss Function	Mean Squared Error
Epochs	100
Batch Size	32

Furthermore, we divided the dataset into training (75%) and testing (25%) subsets, using Mean Absolute Percentage Error (MAPE) as the evaluation metric, calculated as:

$$MAPE=\frac{100}{n}\sum_{i=1}^{n}\frac{\left|y_{i}-{\hat{y}}_{i}\right|}{y_{i}}$$ (11)

where *y*_*i*_ represents the actual incident duration, ${\hat{y}}_{i}$ denotes the predicted incident duration and *n* is the number of data points in the test set.

According to Table 10, when evaluating ML models for incident duration regression using the MAPE metric across various clusters, the LGBM model outperformed all others in Cluster 1, achieving the lowest MAPE score of 0.146. In Cluster 2, the RF model demonstrated the best performance with a MAPE score of 0.153, while in Cluster 3, RF also excelled with a notably low MAPE of 0.010. These findings suggest that model selection should be cluster-specific, with LGBM performing well for Cluster 1, RF for Cluster 2, and RF again for Cluster 3, highlighting the importance of tailoring regression models to specific incident clusters for improved predictive accuracy.

**Table 10:** Comparative analysis of ML models for incident duration regression using MAPE metric.

	Methods							
	RF	XGB	KNN	LR	LGBM	GBDT	ANN	Best Model
**Cluster 1**	0.147	0.150	0.174	0.155	**0.146**	0.150	0.155	LGBM
**Cluster 2**	**0.153**	0.161	0.178	0.164	0.154	0.157	0.162	RF
**Cluster 3**	**0.010**	0.010	0.025	0.026	0.014	0.017	0.022	RF

## Discussion

In this section, we emphasize the critical significance of feature importance for predicting incident duration. By scrutinizing feature importance, we aim to provide decision-makers with actionable insights crucial for improving incident management. This approach empowers decision-makers to strategically allocate resources, optimize response times, and direct their attention to the most influential factors affecting incident duration. Furthermore, our study delves into the exploration of scenarios for boundary clusters. This aspect of our research offers valuable guidance on selecting the appropriate predictive model for incidents that cannot be classified into the main clusters. Essentially, this work enables us to identify and predict incidents that might deviate from the norm and be considered outliers. Finally, the section offers managerial insights, emphasizing unique research findings and implications, to guide decision-makers toward innovative strategies.

### Feature importance

**Fig 5:**
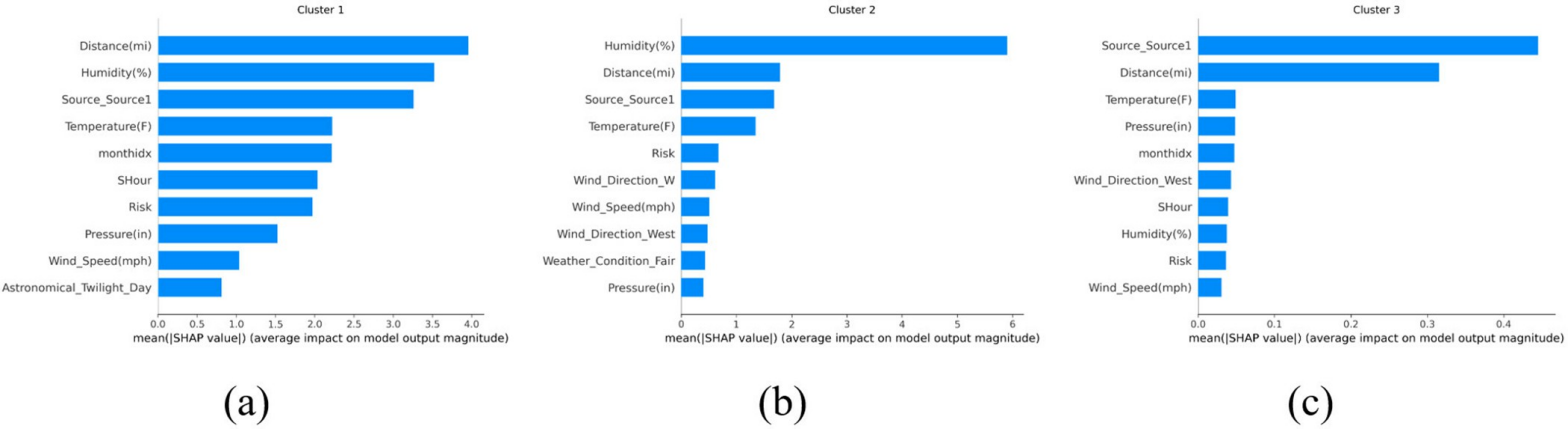
SHAP plots for feature importance in (a) Cluster 1, (b) Cluster 2, and (c) Cluster 3.

In the feature importance analysis section of the paper, SHAP plots were employed to identify the crucial factors influencing incident duration for each cluster. The best-performing models were selected, with LGBM used for Cluster 1 and RF models chosen for Clusters 2 and 3. Fig 5 displays the SHAP plots for each cluster.

The results revealed that our newly introduced risk factor prominently featured among the top influential factors affecting incident duration. The distance of traffic affected by an incident plays a vital role in incident duration by directly impacting congestion levels and the extent of traffic disruption, resulting in longer queues and delays, thereby leading to more complex traffic management and clearance operations. Humidity levels also affect incident duration by influencing road surface conditions. In adverse weather, high humidity can increase road slipperiness, which can raise the likelihood of incidents and lengthen the time needed for incident management and cleanup. Additionally, wind speed and direction are influential variables due to their impact on the dispersion of smoke, debris, and hazardous materials during incidents, potentially complicating firefighting efforts and leading to longer road closures for safety reasons. Moreover, the time of day and the month in which an incident occurs significantly affect incident duration, with factors such as traffic volume and lighting conditions varying throughout the day and across seasons. Incidents during peak traffic hours or adverse weather months, for instance, often experience longer durations due to increased complexity in traffic management and response.

### Regression scenarios for boundary clusters

In this section, we systematically choose suitable models for the boundary clusters and use various scenarios to thoroughly assess their predictive performance. Our study focuses on three specific boundary clusters:

- Boundary of clusters 1 and 2 (BC1-2)
- Boundary of clusters 1 and 3 (BC1-3)
- Boundary of clusters 2 and 3 (BC2-3)

For each boundary cluster, we analyze two distinct scenarios. Each scenario utilizes a predictive model tailored to the specific boundary, with models based on the main clusters. Table 11 offers a clear overview of the assessed scenarios. The details of each scenario are outlined below:

**Scenario 1:** Predicting BC1-2 through the utilization of the model trained on cluster 1 data

**Scenario 2:** Predicting BC1-2 through the utilization of the model trained on cluster 2 data

**Scenario 3:** Predicting BC1-3 through the utilization of the model trained on cluster 1 data

**Scenario 4:** Predicting BC1-3 through the utilization of the model trained on cluster 3 data

**Scenario 5:** Predicting BC2-3 through the utilization of the model trained on cluster 2 data

**Scenario 6:** Predicting BC2-3 through the utilization of the model trained on cluster 3 data

**Table 11:** Scenario evaluation for boundary clusters.

	Methods							
	RF	XGB	KNN	LR	LGBM	GBDT	ANN	Best Model
**Scenario 1**	0.469	0.454	0.529	0.467	**0.453**	0.458	0.465	LGBM
**Scenario 2**	0.264	**0.257**	0.285	0.277	0.265	0.263	0.283	XGB
**Scenario 3**	1.085	1.075	1.154	1.092	**1.030**	1.085	1.058	LGBM
**Scenario 4**	0.475	0.481	0.492	**0.473**	0.478	0.478	0.495	LR
**Scenario 5**	0.652	0.619	0.689	0.605	0.615	0.639	**0.604**	ANN
**Scenario 6**	**0.178**	0.180	0.185	0.187	0.178	0.178	0.185	RF

The table displays the MAPE results for various ML models across different scenarios. Upon interpretation, it is evident that the performance of the ANN is comparable to other traditional models due to the relatively modest dataset size. However, as the dataset scales up, there is potential for ANN to outperform the others. Notably, in the current context, the LGBM model excels, particularly for datasets with medium-sized instances, making it a promising candidate for future considerations.

**Fig 6:**
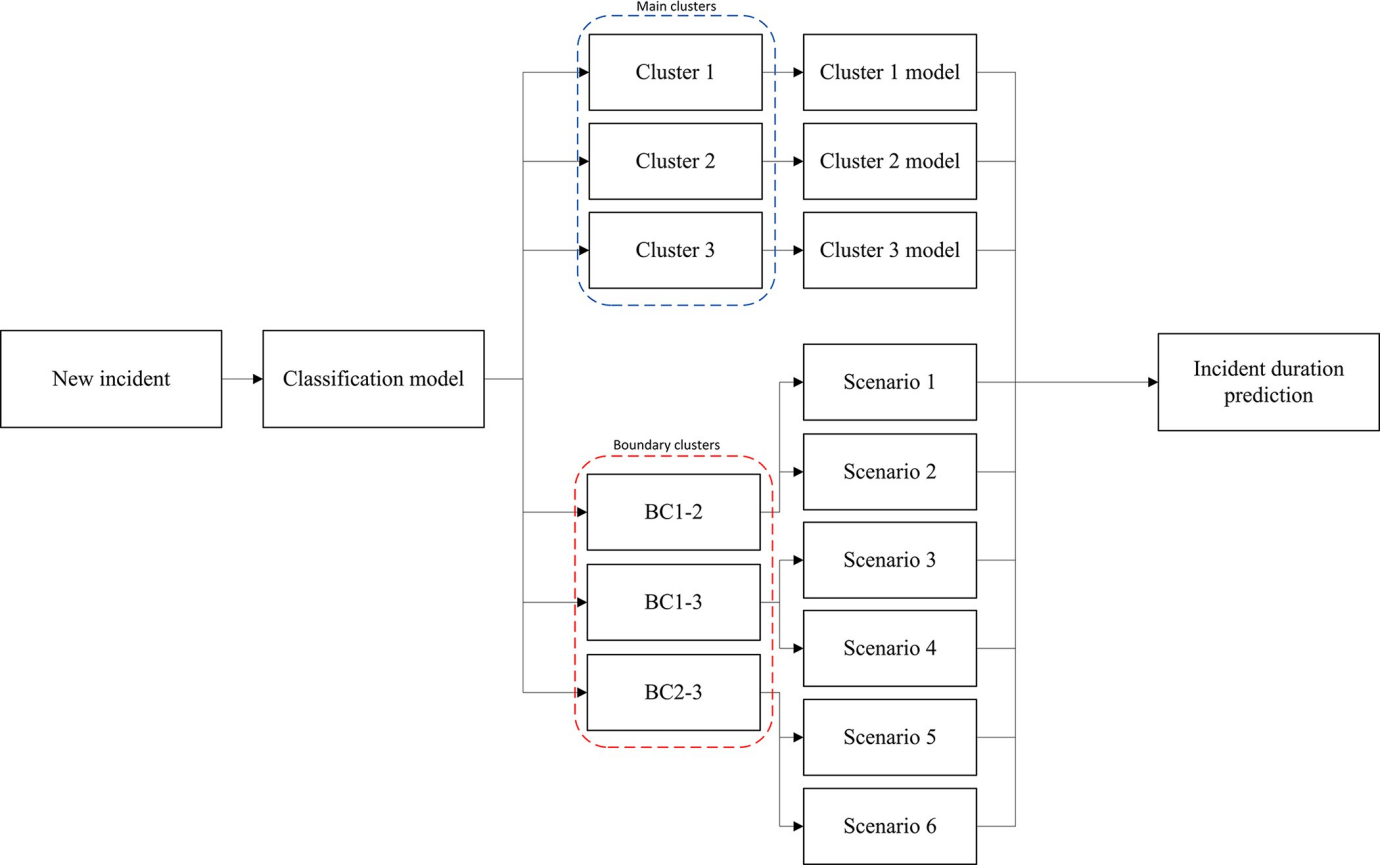
The process of predicting duration for new incident.

To gain a clearer understanding of the predictive process for new incidents, Fig 6 offers a visual representation. This visualization outlines the steps involved after receiving an incident report, with a primary focus on determining the incident cluster, which can be categorized as either part of the main clusters or boundary clusters. Following this categorization, if the incident falls within the main clusters, it is subject to prediction using the main cluster models. However, for incidents classified within the boundary clusters, an additional step involves examining the corresponding scenarios to aid in prediction.

In this paper, we have taken several measures to ensure the reproducibility of the results, which is crucial for validating the effectiveness of the proposed models in real-world applications. We have provided a comprehensive description of the proposed methodology, detailing the data preprocessing steps, feature selection process, and model configuration to enable other researchers to replicate this research study. Additionally, by comparing the proposed framework against well-known traditional methods, we establish a benchmark that others can use in different contexts or with their datasets. Furthermore, we applied the publicly available and comprehensive San Francisco dataset for this study, which not only ensures accessibility for other researchers but also supports direct comparisons and validation of the results. Finally, we have meticulously documented all the model parameters, including hyperparameters and training configurations, to facilitate the replication of the proposed model’s training process under similar conditions. These steps collectively enhance the reproducibility of the results and support its application in real-world scenarios.

### Managerial insights

This study offers novel insights into improving the traffic incident duration prediction by addressing key gaps in the existing literature, particularly focusing on boundary clusters and street-specific risk factors. The managerial implications of these contributions are significant, particularly for urban traffic management authorities and policymakers seeking to enhance the efficiency and responsiveness of their systems.

One of the core contributions of this study is the integration of street feature risk factors into traffic incident prediction. Traditional models have often overlooked these risk factors, leading to less accurate predictions. By incorporating the RPN concept, this paper provides a more granular understanding of how specific street characteristics influence incident durations. This approach enables the managers to allocate resources more effectively, anticipating incident duration based on the unique risks associated with different streets. This improved prediction accuracy could lead to better planning for incident response, reducing delays and improving traffic flow. Furthermore, this research challenges the traditional, rigid classification of traffic incidents, often limited by the FHWA definition. Applying flexible clustering techniques, including fuzzy clustering, in this study allows for a more dynamic categorization of incidents, accommodating the complexities and variations found across diverse datasets and locations. This flexibility provides traffic managers with a more adaptive tool for incident management, as it allows for the classification of incidents based on a broader set of features rather than fixed categories. By doing so, it enables more precise targeting of intervention strategies and resource allocation. Another key contribution is the use of MCDM techniques to rank incident clusters based on their potential impact on traffic flow. The inclusion of fuzzy MCDM offers a more sophisticated assessment of the probabilistic nature of incidents, enabling better prioritization of responses. Traffic managers can leverage this approach to identify which incidents will have the most severe consequences on traffic flow, allowing for quicker, more informed decision-making. It could be noted that one of the most unique aspects of this paper is the introduction of scenario-based predictive modeling for boundary clusters. Unlike conventional models that typically focus on incidents within clearly defined clusters, this study provides a systematic approach for predicting incidents that lie at the boundaries between multiple clusters. This scenario-based approach ensures that predictions in these complex areas are more accurate, offering a flexible framework for addressing incidents that do not fit neatly into predefined categories. This is particularly valuable for cities with complex traffic networks, where incidents often span multiple traffic flow patterns. By incorporating this approach, traffic management systems can become more adaptable, accurately predicting the duration and impact of incidents in boundary areas where traditional models fall short.

In summary, this study provides managers with a comprehensive, flexible, and data-driven approach to traffic incident prediction. By addressing key gaps in the current literature and offering innovative solutions, this paper differentiates itself from prior research, providing actionable insights that can be directly applied to improve urban traffic management. These contributions not only enhance the precision of traffic incident duration predictions but also offer practical tools for better resource allocation, optimized traffic flow, and more effective incident response.

## Conclusion

Incident management programs are a crucial component of traffic management in both urban and rural areas. Their primary objectives are to minimize the negative impacts on traffic congestion, swiftly restore full road network capacity after an incident, and reduce delays for commuters and travelers. Accurate predictions of incident durations are essential for optimizing resources and minimizing disruptions in traffic management. These predictions enable efficient deployment of response teams and strategic traffic rerouting, ultimately reducing congestion and enhancing safety. Moreover, a comprehensive understanding of various incident types facilitates the implementation of preventive measures and the development of strategies to mitigate their impact on road networks. In this paper, we present a comprehensive framework that combines Machine Learning (ML) approaches and Multiple Criteria Decision-Making (MCDM) methods to accurately predict the duration of traffic incidents and enhance our understanding of their impact on traffic flows. We introduce a novel feature called "Risk," derived from the Risk Priority Number (RPN) concept, to highlight the crucial role of incident location in both the occurrence and prediction of incidents. This feature highlights the need for a detailed evaluation of streets based on calculated risk to reduce the likelihood of future incidents. In addition, we applied the fuzzy clustering method to redefine incident categorization, creating a clear policy for identifying boundary clusters that need further modeling and testing under different scenarios. Fuzzy clustering is employed to distinguish confidently categorized incidents from those that do not fall into a specific cluster, necessitating additional consideration for each category. Each main cluster undergoes an MCDM process to achieve better visions into their distinctions and offer valuable managerial visions. Within the MCDM section, we determine the significance of each cluster based on their influence on traffic flow and their potential need for additional resources and attention. Finally, we employ a combination of traditional ML models and Deep Learning (DL) models to conduct both classification and regression processes. We employ Artificial Neural Network (ANN), Random Forest (RF), XGBoost (XGB), K-Nearest Neighbors (KNN), Logistic Regression (LR), Light Gradient Boosting Machine (LGBM), and Gradient Boosted Decision Trees (GBDT) for both classification and regression tasks. These regression models are compared using the MAPE metric. Significantly, we will predict incidents in the boundary clusters by applying the relevant scenarios. Through a rigorous examination of feature importance in the prediction process, utilizing high-performing models and SHAP plots, we establish that our newly introduced feature, “Risk”, emerges as a crucial factor in determining incident duration. Moreover, the features such as distance, humidity, and time of day significantly influence incident duration. However, it is essential to acknowledge several limitations inherent in this study. One notable limitation relates to the datasets, which may lack comprehensive information about specific incidents. This deficiency extends to missing data related to critical factors such as vehicle type, road classification, and driver information, posing challenges for conducting in-depth analyses. Furthermore, the reliability of the dataset is susceptible to issues associated with data quality, encompassing inaccuracies and inconsistencies in reporting. Variations in driving regulations across different regions can also impede the generalizability of our analyses. Future research endeavors should consider the impact of varying regulations on incidents in diverse geographical areas. Moreover, building upon the findings of our study that underscore the influence of the "Risk" feature on incidents in each street, future investigations could aim to explore the concept of risk from novel perspectives and dimensions.

## Supporting information

S1 File (DOCX)
