# Supplementary material for: Incident duration prediction through integration of uncertainty and risk factor evaluation: A San Francisco incidents case study
Source: PLoS One. 2025 Jan 2;20(1):e0316289. doi: 10.1371/journal.pone.0316289 (PMC11694969; doi:10.1371/journal.pone.0316289)
Supplement: S1 File — (DOCX) [file pone.0316289.s001.docx]

# Appendix A

The procedure for the FCM algorithm is outlined as follows:

**Step 1:** Initialize$c$ and $m$

where $c$ represents the number of clusters and $m$ signifies the fuzziness parameter that controls the extent of fuzziness.

**Step 2:** calculate the membership degree $u_{ij}$, which signifies the degree to which data point $x_{i} (i=1, 2,\ldots, N)$ belongs to cluster $j (j=1, 2, ..., c)$:

| $u_{ij}=\frac{1}{\sum_{k=1}^{c} \left( \frac{ⅆ_{ij}}{ⅆ_{ik}} \right)^{\frac{2}{m-1}}}$ | (A1) |
| --- | --- |

where $d_{ij}$ is the distance between $x_{i}$ and cluster centroid $j, d_{ik}$ is the distance between data point $x_{i}$ and cluster centroid $k$ and $m$ is the fuzziness parameter.

**Step 3**: For each cluster $j$, the new centroid $c_{j}$ is updated:

| $c_{j}=\frac{\sum_{i=1}^{N} u_{ⅈj}^{m}x_{i}}{\sum_{i=1}^{N} u_{ⅈj}^{m}}$ | (A2) |
| --- | --- |

**Step 4:** Establish a termination criterion, which can be in the form of a maximum iteration count or a threshold indicating centroid convergence. Continue the iterative process by repeating steps 2 and 3 until the termination criterion is satisfied.

# Appendix B

Fuzzy Vikor steps is outlined as follows:

**Step 1:** Start by identifying the objectives of the decision-making process, defining the problem scope, and arranging the decision-making group. Additionally, define and describe a finite set of relevant attributes for the decision-making process.

**Step 2:** The process involves obtaining aggregated fuzzy weights for criteria and aggregated fuzzy rankings for alternatives from decision makers, resulting in the creation of a fuzzy decision matrix. To determine the aggregate fuzzy rating (represented as $\tilde{x}_{ij}$) of alternatives concerning each criterion, the following calculation is performed:

| $\tilde{x}_{ij}=(x_{ij1},x_{ij2},x_{ij3})$ | (B1) |
| --- | --- |

where $x_{ij1}=min\{x_{ij1}{\}}_{k}$​, $x_{ij3}=max\{x_{ij3}{\}}_{k}$, and the aggregated fuzzy weights (represented as $\tilde{w}_{j}$) for each criterion can be computed as:

| $W=(\tilde{w}_{1},\tilde{w}_{2},\tilde{w}_{3})$ | (B2) |
| --- | --- |

where $w_{j1}=\min\left\{ w_{jk1} \right\}_{k}, w_{j2}=1/{k\sum_{k}^{K} w_{jk2}}$ and $w_{j3}=\max\left\{ w_{jk3} \right\}_{k}.$

A decision matrix denoted as D, characterized by its dimensions of m rows and n columns, is formally defined by Equation (B3):

| $D= A_{i}\left[ \begin{matrix} A_{i}\tilde{x}_{11} & \ldots& \tilde{x}_{1j} & \ldots& \tilde{x}_{1n} \\ A_{i}\tilde{x}_{i1} & \ldots& \tilde{x}_{ij} & \ldots& \tilde{x}_{in} \\ A_{i}\tilde{x}_{m1} & \ldots& \tilde{x}_{mj} & \ldots& \tilde{x}_{mn} \end{matrix} \right]$ | (B3) |
| --- | --- |

where the variables $x_{ij}$ can exist as either precise or fuzzy values. In the case of $x_{ij}$ being fuzzy, it is expressed using a triangular fuzzy number (TFN) denoted as $\tilde{x}_{ij}$ with parameters $a_{ij}, b_{ij}$, and $c_{ij}$. Describing the fuzzy weights is achieved through Equation (B5):

| $W=\left( \tilde{w}_{1}\ldots\tilde{w}_{j}\ldots\tilde{w}_{n} \right);\tilde{w}_{j}=\tilde{\alpha}_{1},\tilde{\beta}_{j},\tilde{\chi}_{n}$ | (B4) |
| --- | --- |

**Step 3**: To convert the fuzzy decision matrix and fuzzy weight of each criterion into precise values, a commonly used approach is the Center of Area (COA) method. When applying the COA method to calculate the BNP (Best Numerical Performance) value for triangular fuzzy performance scores represented as $\tilde{h}_{ai}=(lh_{ai},{mh}_{ai},{uh}_{ai})$, the following steps are taken:

| $x_{ai}=lh_{ai}+\frac{\left( uh_{ai}-lh_{ai} \right)+\left( mh_{ai}-lh_{ai} \right)}{3},\forall a$ | (B5) |
| --- | --- |

**Step 4:** Identify the best $f_{j}^{*}$ and the worst $f_{j}^{-}$ values for all criterion ratings, where $j$ ranges from $1 to n$.

| $f_{j}^{*}=\max\left( f_{ij} \right)$ | (B6) |
| --- | --- |
| $f_{j}^{-}=\min\left( f_{ij} \right)$ | (B7) |

where $f_{j}^{*}$ represents the positive ideal solution for the $j$th criterion, and $f_{j}^{-}$​ represents the negative ideal solution for the same criterion. When combining all $f_{j}^{*}$​ appropriately, the result will yield an optimal combination that achieves the highest scores comparable to those of $f_{j}^{-}$.

**Step 5:** Calculate the $S_{i}$ and $R_{i}$ values for $i$ ranging from $1 to m$ by applying Equations (19) and (20).

| $S_{i}=\sum_{j=1}^{n} W_{j}\left[ \frac{f_{j}^{*}-f_{ij}}{f_{j}^{*}-f_{j}^{-}} \right]$ | (B8) |
| --- | --- |
| $R_{i}=max_{j}\left[ W_{j}\left( \frac{f_{j}^{*}-f_{ij}}{f_{j}^{*}-f_{j}^{-}} \right) \right]$ | (B9) |

where $S_{i}$ signifies the rate of distance of the $i$th alternative to the positive ideal solution, and $R_{i}$ represents the rate of distance of the $i$th alternative to the negative ideal solution, we also have $w_{j}$ denoting the weights attributed to criteria, reflecting their respective levels of relative importance.

**Step 6:** Calculate the $Q_{i}$ values for $i$ ranging from $1 to m$, utilizing Equation (21):

| $Q_{i}=v\frac{S_{i}-S^{*}}{S^{-}-S^{*}}+(1-v)\frac{R_{i}-R^{*}}{R^{-}-R^{*}}$ | (B10) |
| --- | --- |

where $S^{-}={max}_{i}S_{i}, S^{*}={min}_{i}S_{i}, R^{-}={max}_{i}R_{i}, R^{*}={min}_{i}R_{i}$. Additionally, $v$ denotes the weight assigned to the strategy based on "the majority of criteria" or "the maximum group utility." In this context, $v$ is supposed to be 0.5.

**Step 7:** Order the alternatives based on the $Q_{i}$ values determined in step (6).
